# A Comprehensive Insight into the Phytochemical, Pharmacological Potential, and Traditional Medicinal Uses of *Albizia lebbeck* (L.) Benth.

**DOI:** 10.1155/2022/5359669

**Published:** 2022-04-21

**Authors:** Acharya Balkrishna, Sakshi, Mayur Chauhan, Anurag Dabas, Vedpriya Arya

**Affiliations:** ^1^Patanjali Yogpeeth Trust, Prasauni-03, Birgunj-Kalaiya Road, Bara, Province-2, Nepal; ^2^Patanjali Herbal Research Department, Patanjali Research Institute, Haridwar 249405, Uttarakhand, India; ^3^University of Patanjali, Patanjali Yogpeeth, Haridwar 249405, Uttarakhand, India

## Abstract

**Background:**

*Albizialebbeck* is a deciduous tree having tremendous medicinal utilities, for example, respiratory, skin, gastrointestinal, oral disorders, eye, urinary, genital, anorectal, inflammatory, and neurological disorders, and venereal diseases. Several studies have been undertaken on the medicinal and traditional values of *A. lebbeck*.

**Objective:**

The detailed information about its medicinal uses and pharmacological implications is highly scattered and distributed in different data sources. Hence, the study was conducted to supply an inclusive review of its ethnomedicinal uses, phytochemicals, and the available pharmacological attributes supporting its efficiency in traditional medicine.

**Method:**

Literature surveys were conducted on this medicinal plant *via* search engines like Google Scholar, PubMed, and Science Direct, and obtained information up to December 2020 has been assessed and analyzed for this study.

**Results:**

Systematic investigation revealed that *A. lebbeck* consists of various phytochemicals, including major alkaloids, flavonoids, saponins, and terpenoids. Its crude extract, fraction, and bioactive compounds exhibited potent adulticidal, antiallergic, anticancer, anticonvulsant, antidiabetic, antidiarrheal, anti-inflammatory, antimicrobial, antinociceptive, antioxidant, antiparasitic, antipyretic, antivenom, estrogenic, neuroprotective, nootropic, ovicidal, and wound healing activities.

**Conclusions:**

This study proposes that *A. lebbeck* remains a rich source of phytochemicals with various biological activities which possess outstanding therapeutic benefits to humanity across the world. However, studies are required to estimate the potential side effects. Moreover, mechanistic physiognomies of the isolated compounds with known bioactivities are quite limited; thus, forthcoming research needs to focus on the mechanisms of these active phytochemicals to facilitate their potential enrolling for drug discovery.

## 1. Introduction

**Figure 1:**
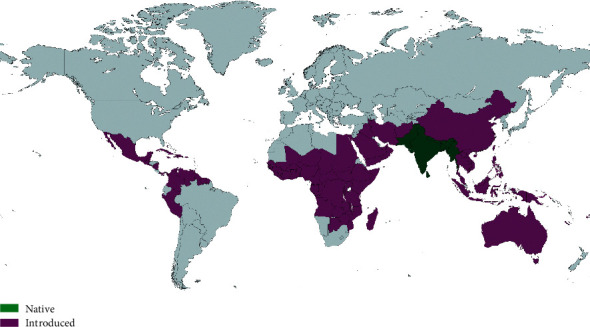
Global distribution of medicinal species *Albizia lebbeck* (L.) Benth. (created with mapchart.net).

Medicinal plants and their derived natural products have long served as the primary healthcare requirements of millions of populations for centuries. Among these medicinal plants, many plants have been scientifically documented and validated for their exceptional medicinal efficacy. The genus *Albizia* comprises 150 taxonomically accepted species, which are widely distributed in Asia, Africa, and Australia, as well as tropical and subtropical America [1]. *Albizia lebbeck* mainly grows in the Indian subcontinent and Myanmar (Burma) and is also widely distributed in Western and Southeast Asia, Australia, Northern and West Africa, throughout the Caribbean, Central America, and the northern and eastern regions of South America (Figure 1) [2]. This species is reported to have incredible therapeutic properties, and it is utilized in several countries throughout the world to treat a variety of diseases and disabilities. The plant has been traditionally used against various diseases such as ulcers, night blindness, respiratory disorders, skin disorders, snake, bite, piles, and leprosy [3–5]. It is also used against gonorrhea, scorpion bite, gum problems, cough, pharyngitis, and so on [6–8]. In Sanskrit nomenclature, it is known as Sirisha, Bhandi, and Sirisa, while it is also entitled in many other languages throughout the world, for example, Acacia amarilla, cabellos de ángel, and lengua de mujer in Spanish; Bois noir and Viellefille in Franz; Darash in Urdu; Karuvagei and Vagei in Tamil; Khago and Ka se in Thai. In Burmese, it is spelled Kokko; Lebbek, siris tree, and woman's tongue tree in English; Mara in Sinhalese; Sarin and Shrin in Punjabi; Siris, SIrish, and Sirisha in Bengali; Siris and Sirisha in Hindi; Sultanaulasjar in Arabic; and Tekik in Javanese [2, 9].

It is a deciduous tree that is mostly found in the garden or along the roadside and grows from sea level to 1500 m elevation, attaining height up to 18 m. *A. lebbeck* contains numerous phytochemicals related to alkaloids, anthraquinones, essential oils, flavonoids, glycosides, phenolics, phytosterol, saponins, steroids, and triterpenoids [9–13]. According to various pharmacological studies, this species exhibited excellent antinociceptive, anti-inflammatory [11], anticancer [9], antimalarial [14], antiallergic [15], antihyperglycemic [16], antidiabetic [17, 18], wound healing [19], nootropic [20], and neuroprotective activities such as anti-Parkinson's and anti-Alzheimer activities [12, 21]. Furthermore, zinc oxide nanoparticles synthesized from *Albizia lebbeck* stem bark extract caused concentration-dependent organoprotective effect by changing mean body weight, alanine aminotransferase, serum alkaline phosphatase, urea, creatinine, bilirubin, protein, globulin, albumin, total cholesterol, triacylglycerol, and low- and high-density lipoprotein [22]. Other than its medicinal applicability, it is also used for reforestation of degraded sites, fuelwood plantations, and agroforestry systems in Asia [2].

This species contains a huge number of phytochemicals, out of which several phytochemicals have excellent medicinal properties and also showed tremendous pharmacological activities. There are a couple of compounds that have been exposed to pharmacological examinations and deficiently summed up with dispersed and scant data accessible on traditional uses. Additionally, there has been a lack of information that relates the pharmacological attributes of this plant to its ethnomedicinal applications. Likewise, patented formulations and safety profiles have been inadequately explored.

Even though many studies have been published on the biological activity of *A. lebbeck* extracts and their phytoconstituents [23–25], none of the reviews has been published with comprehensive information on pharmacological activities and elaborative insights of countrywise medicinal uses as well as different medicinal systemwise therapeutic potential. This prompted us to write this study, which covers botanical description, taxonomy, geographic distribution, medicinal usage, phytochemistry, and pharmacological qualities of *A. lebbeck*. The obtained information on phytochemicals, therapeutic uses, and pharmacological credits would optimistically assist the scientific community in planning safe tests that incorporate bioactive mixtures.

## 2. Materials and Methods

For this paper, an inclusive literature search was conducted up to January 2021. To identify appropriate statistics on the botanical description, traditional medicinal uses, phytochemistry, and pharmacological activities of *A. lebbeck*, information was retrieved from various resources, including Google Scholar, Science Direct, PubMed, and literature books. The keywords used for the database were “*Albizia lebbeck*,” “Medicinal Uses,” “Traditional Uses,” “Botany,” “Chemical Constituents,” “Pharmacology,” and “Biological Activities” with Boolean operators. Database that was unsuccessful in meeting the inclusion and quality criteria required in traditional uses, phytochemistry, and pharmacological attributes was excluded. The scientific name of the plant was authenticated by different databases like “the plant list” and “plants of the world online” (http://www.plantsoftheworldonline.org/; http://www.theplantlist.org/).

## 3. Botanical Description

**Figure 2:**
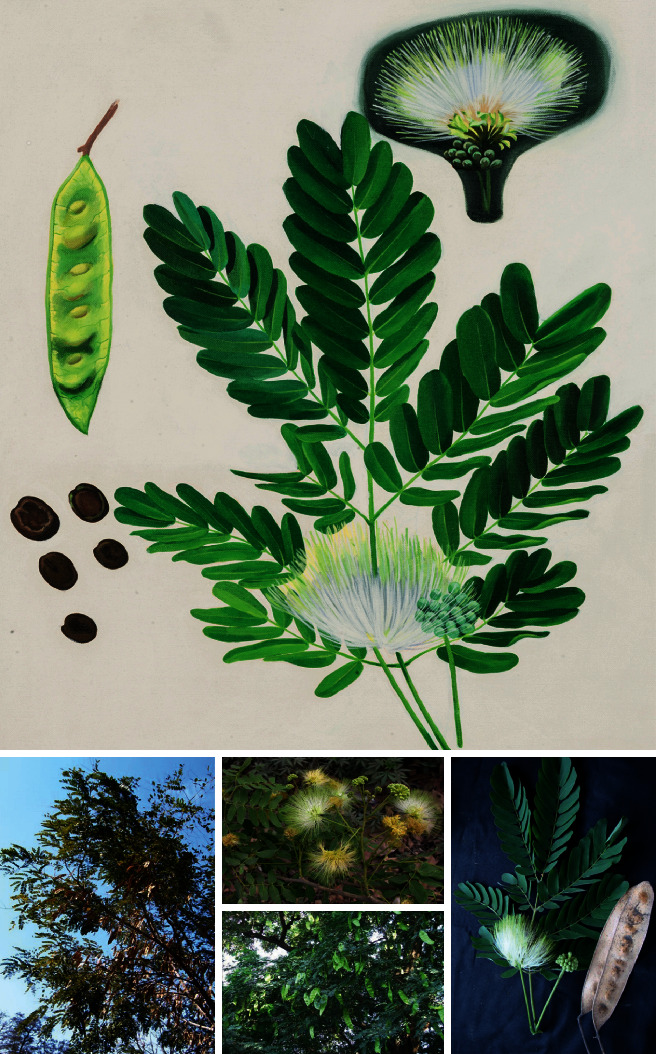
Leaves, flowers, and pods of *Albizia lebbeck* (source: Patanjali Herbal Museum).

*Albizia lebbeck* grows as a deciduous tree with a length up to 18 m and a straight bole. Its bark is brownish-gray in color. The leaves of the plant are bipinnate, which are alternately arranged on the smooth, green twigs. The leaves turn a deep yellow color before falling during the dry season. The inflorescence is of corymb type with 30–40 flowers. Flowers are dimorphic, puberulent, and fragrant white to greenish-yellow in color. Calyx and Corolla are funnel-shaped; their pod is pale, flat, and straw-colored and remains on trees after a long-time of ripening. Seeds are brown, ellipsoidal (4–12) ca. 10 × 6–7 mm, and their pleurogram is parallel to margins of the seed [2]. The picture of *A. lebbeck* plant and its different parts is shown in Figure 2.

## 4. Traditional Medicinal Uses

**Figure 3:**
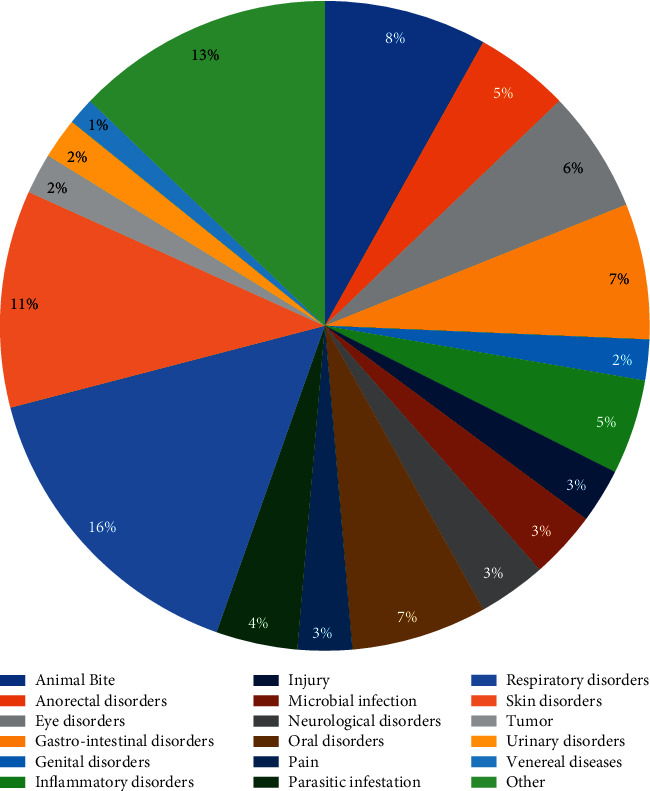
Percentage of reported ethnomedicinal uses of *A. lebbeck* against myriad diseases.

**Figure 4:**
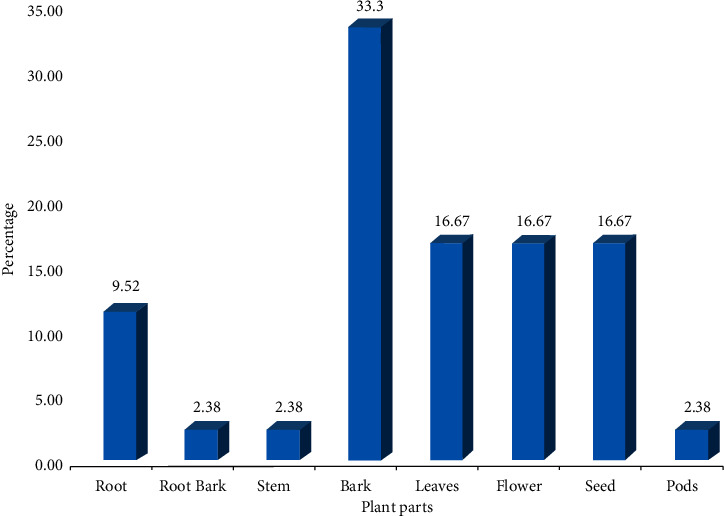
Parts usage (%) of *A. lebbeck* reported for various ethnomedicinal uses.

*A. lebbeck* has been used in various countries of Africa, Asia, and Australia for the prevention of scabies, lung ailments, piles, bronchitis, abdominal tumors, cough, eye disorders, and so on. It is recommended in several medicinal systems, for example, Ayurveda, Sidha, and Unani medicine (Table 1) [11, 14, 31]. It has been used in numerous traditional uses; among them, it is mostly used in the treatment of respiratory disorders with 16%, skin disorders with 11%, and gastrointestinal disorders and oral disorders with 7% (Figure 3). In all these ethnomedicinal and traditional entities, the plant is ordinarily used to treat asthma, bronchitis, diarrhea, and gum inflammation with 4.88%, piles with 4.27%, parasitic infestation and snakebite with 3.66%, ulcer, scorpion sting, leprosy, and boils with 3.05%, and abdominal tumor, arthritis, cough, dysentery, night blindness, and poisoning with 2.44% in various countries. All plant parts, including root, leaves, flowers, bark, and seed, are useful in Indian traditional medicine in the treatment of several health ailments, for example, allergies, asthma, bronchitis, arthritis, fractures, gingivitis, gum inflammation, toothache, hemorrhage, leprosy, leukoderma, malaria, night blindness, scorpion sting, snakebite, and syphilis [10, 15, 26]. The bark is the most used plant part with 33.33% usage, followed by leaves, flower, seed (16.67%), root (9.52%), root bark, stem, and pods (2.38%) (Figure 4). *A. lebbeck* has many therapeutic values such as astringent, pectoral, rejuvenation, and tonic [31].

According to the Ayurvedic Pharmacopoeia of India (2016), the stem bark possesses therapeutic uses such as *Pama* (eczema), *Kustha* (leprosy), *Kandu* (pruritus), *Visarpa* (erysipelas), *Kasa* (cough), *Vrana* (ulcer), *Sotha* (inflammation), *Svasa* (dyspnea), *Musaka Visa*, *Sita Pitta* (urticarial), *Raktadusti* (hypertension), *Pinasa* (catarrh), *Vismajvara* (irregular fever), *Pratisyaya* (common cold), *Sarpdansa* (snakebite), *Visadusti*, *Suryavarta* (migraine), Ardhavabhedaka (headache in half side of the head), *KrmiRoga* (worm infestation), and *Netrabhiasanda* (conjunctivitis). It retains various properties and actions; for example, *Rasa* is *Madhura* (sweet), *Katu* (pungent), *Tikta* (bitter), and *Kasaya* (astringent); *Guna* is *Laghu* (lightness); *Virya* and *Vipaka* are *Anusna* (lukewarm) and *Katu* (pungent), respectively; and *Karma* is *Sothahara* (alleviate swelling), *Tridosahara* (pacifies the three doshas), *Visghna* (neutralizing poison), *Tvagdosa* (skin disease), and *Varnya* (skin lightening). *A. lebbec*k has been widely used as an ingredient in several polyherbal formulations, for example, *Vajraka Taila*, *Dasanga Lepa*, *Ayakrti*, *Devadarvarista*, and *Brhanmaricyadi Taila* [32]. Bark and flowers are helpful in arthritis, and they are used in the Siddha system [18]. About 5–6 g of fresh leaves and 4–5 g of misree (refined sugar) in 1 glass of water, ground in a clay pot, can be taken 3 times a day to prevent tuberculosis. Fresh leaves are chewed, and then their extract from the mouth is poured into the eyes after filtration with a clean thin piece of cloth to soothe the reddishness of the eyes. 10–15 g of seeds is ground in a clay pot with water and consumed twice a day after filtration for the cure of boils by Sindh Indigenous people [33]. Moreover, the Bhils tribes used powder of crushed stem bark that can be applied on boils and pimples and paste of leaves and bark to cure insect bite and scorpion sting [8].

The stem bark paste is applied on ulcer and flower decoction and leaves for gargling to cure weak and spongy gums and chronic pharyngitis by the Meena tribe [8]. The Zulu tribes from Africa use bark and roots in the treatment of scabies, inflamed eyes, piles, and bronchitis [11]. In Tibetan traditional medicine, it is recommended in the treatment of kapha, pitta, poisoning, erysipelas, and ulcer [34]. In Taiwan, it is used as an anthelmintic, diuretic, stimulant, and tonic [35]. The people of Tamil Nadu use plants to fix bone fractures. The tribal communities in Himachal Pradesh and Kashmir use plants to relieve inflammation [28]. It is commonly called Shirish, Koroi, and Parrot tree in Bangladesh and has been used by the local people in the treatment of ophthalmia. Additionally, its barks and seeds are used as astringent and are given in piles, diarrhea, toothache, and gum problems. Further, bark and leaf decoctions are recommended against bronchial asthma and other allergic disorders [36]. Moreover, saponins of *A. lebbeck* have been reported to be used in Alzheimer's and Parkinson's disease treatment [37]. The ethnomedicinal uses, including data from various countries and medicinal practices of *A. lebbeck,* are given in Table 2.

## 5. Phytochemistry

Phytochemical studies of *A. lebbeck* have exposed the presence of various chemical constituents, including alkaloids, phenols, flavonoids, saponins, phytosterols, and terpenes [14]. Besides, seeds are good source of protein 2.272%, lipids 0.27%, fatty acid (linolenic acid, oleic acid, palmitic acid, and steric acid), tetradecane, hexadecane, phytol, nonadecane, eicosane, vitamin E, stigmastadiene, and octadecane [21, 45]. Complex triterpenoid saponin, that is, 21-[(2E,6S)-6-[6-deoxy-4-O-[(2E,6S)-6-hydroxy-2-(hydroxymethyl)-6-methyl-1-oxo-2,7-octadienyl]-[(*β*-D-glucopyranosyl) oxy]-2-(hydroxymethyl)-6-methyl-1-oxo-2,7-octadienyl]-[(*β*-D-glucopyranosyl)oxy]-2,6-dimethyl-1-oxo-2,7-octadienyl]oxy]-16-hydroxy-3-[[O-*β*-D-xylopyranosyl-(1 ⟶ 2)-O-*α*-L-arabinopyranosyl-(1 ⟶ 6)-2-(acetylamino)-2-deoxy-*β*-D-glucopyranosyl]oxy]-(3*β*, 16*α*, 21*β*)-olean-12-en-28-oic acid O-*α*-L-arabinofuranosyl-(1 ⟶ 4)-O-[*β*-D-glucopyranosyl-(1 ⟶ 3)]-O-6-deoxy-*α*-L-mannopyranosyl-(1 ⟶ 2)-*β*-D-glucopyranosyl ester, is isolated from the bark [46]. Other than that, leaves contain essential oil in which 2-pentylfuran (16.4%), (E)-geranyl acetone (15.46%), (E)-*α*-ionone (15.45%), and 3-Octanone (11.61%) are abundantly found [11]. The present review suggests that the majority of phytochemicals contained in *A. lebbeck* should be explored and isolated from its bark and seeds, and additionally, other parts should be investigated too in the wake of the maximum utility of this plant to mankind.

**Figure 5:**
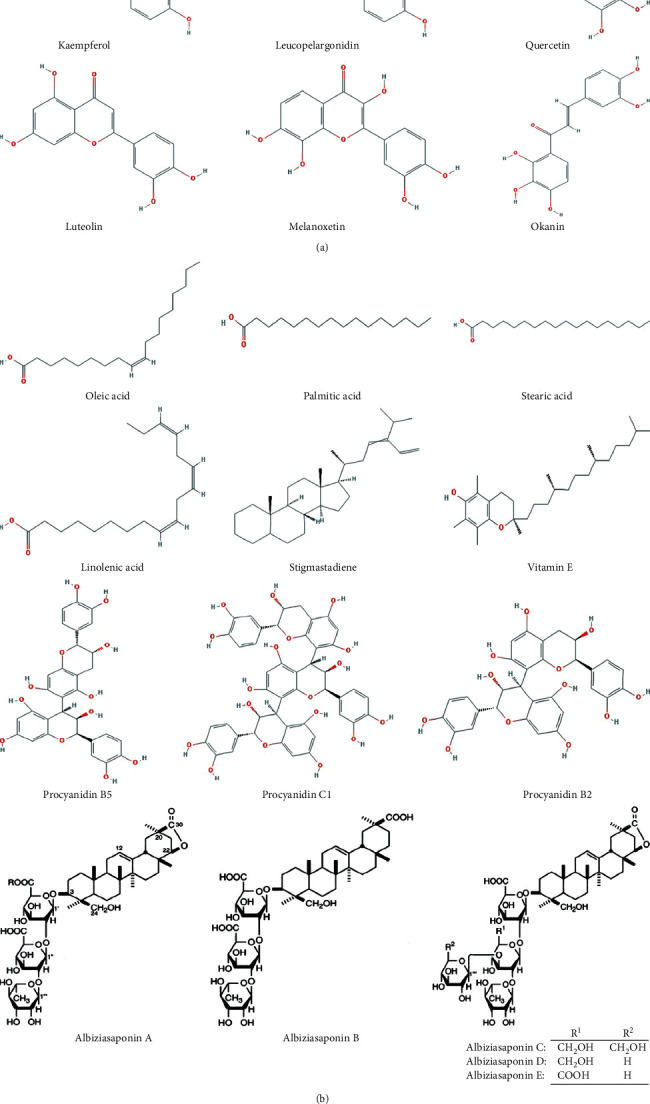
Molecular structure of various phytochemicals extracted from different parts of *Albizia lebbeck*.

The bark contains albiziasaponins (A–E) and lebbeckoside C, which possesses anticancer activity [9, 38]. Lebbeckosides A-B isolated from root showed an inhibitory effect on high-grade human brain tumor cells [31]. However, the seed contains lebbeckalysin (hemolysin), which possesses potent antitumor and antimicrobial effects [47]. Flavonoids (geraldone, luteolin, and isookanin) were isolated from the bark having the capability of inhibiting the *α*-glucosidase and *α*-amylase activity [17]. Among reported chemical compounds, 45 bioactive molecules have been discussed in the pharmacological section. These studies suggested that most of the phytochemicals have been isolated from bark and seeds, and other parts are still needed to be explored. Plenty of molecular structures of various phytochemicals are procured from PubChem, and their detailed information is given in Table 3 and Figure 5.

## 6. Pharmacological Activities

**Figure 6:**
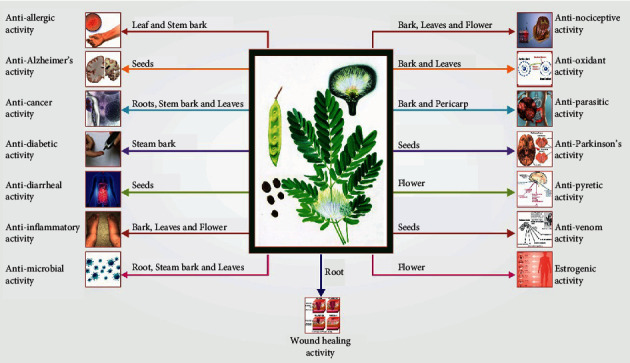
Different parts of *Albizia lebbeck* used for several pharmacological investigations.

Several pharmacological studies showed that extracts/fraction/compounds of leaves, bark, and flower of *Albizia lebbeck* (L.) Benth exhibited significant antiallergic activity, anticancer, anticonvulsant, antidiabetic, anti-inflammatory, antimicrobial, antinociceptive, antioxidant, antiparasitic, antivenom, neuroprotective, nootropic, antipyretic, antidiarrheal, ovicidal, adulticidal activity estrogenic, and wound healing activities. The foremost pharmacological attributes, extract/fraction/compound extracted from different parts of the plant, investigational doses, experimental models, and their results have been given in Figure 6, and pharmacological activities are also described as follows.

### 6.1. Antiallergic Activity

Ethanolic extract (200 mg/200–250 gm b. w., p.o.) of *A. lebbeck* stem bark exhibited excellent antiallergic activity in toluene-2,4-diisocyanate- (TDI-) sensitized allergy model Brown Norway rats and HeLa cells expressing endogenous H1R with a significant decrease in the numbers of sneezing, nasal rubbing, and mRNA expression which have been found to elevate TDI-induced H1R and HDC, although the least doses of extract (0.1 to 10 *μ*g/ml) also reduced PMA- or histamine-induced upregulation of H1R mRNA in HeLa cells [48]. Besides, catechin present in the ethanolic extract from *A. lebbeck* bark showed potent activity by modulating histamine release and cytokine expression. *In vitro*, chloroform, methanol, and water extracts of leaf and bark showed a significant mast cell stabilizing effect with 19.71–59.69% against compound 48/80 [15, 51].

### 6.2. Anticancer Activity

**Figure 7:**
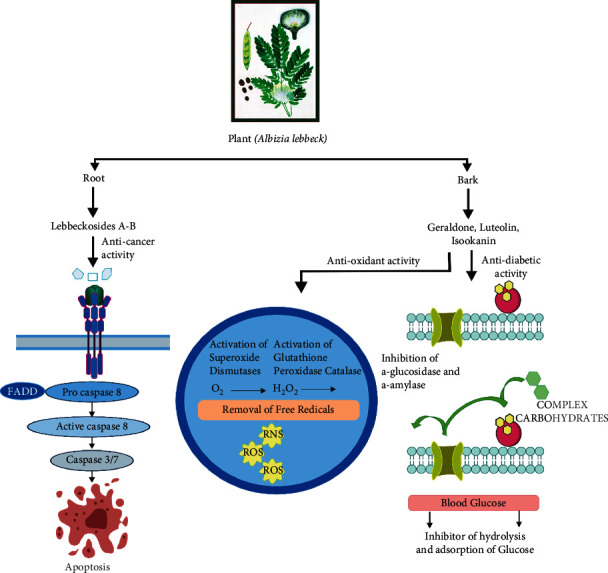
Mechanistic representation of different phytochemicals extracted from *A. lebbeck*.

Bark and leaves of *A. lebbeck* showed a potent anticancer effect from diverse cell lines. A saponin-rich fraction from the bark of *A. lebbeck* exerted antiproliferative activity *via* MTT assay in human breast cancer cell line MCF-7 by inhibiting the growth with IC_50_ 1 µg/ml and inducing apoptosis at 10 µg/ml by promoting activation of caspases 3 and 8. Furthermore, in shell-less chick embryo culture assay, there was a significant (*p* < 0.05) reduction in the number of extremities, nodes, junctions, and total branches length between 0 and 3 hr and 0–6 hr of drug exposure (0.1, 0.5, and 1 *μ*g/ml) and elevation of chromosomal aberration observed [40]. In another study, lebbeckosides A and B isolated from the root showed significant cytotoxic activity against U-87 MG, TG1 high-grade human brain tumors cells with IC_50_ 3.46, 1.36, and 2.10, 2.24 *μ*M, respectively [31]. The isolated compounds lebbeckosides A and B are responsible for initiating apoptosis in the cancerous cell by the activation of caspase 8 (Figure 7). Apart, crude methanol extract from leaves exerted a cytotoxic effect on hepatocarcinoma (HepG2) cancer cell line with IC_50_ 24.03 *μ*g/ml [52]. In another study, gold nanoparticles isolated from aqueous leaf extract of *A. lebbeck* showed cytotoxicity against HCT-116 colon cancer cells with IC_50_ 48 mg/ml and also induced apoptosis by increased ROS production, decreased ΔΨm, apoptotic morphological changes by AO/EtBr, and altering pro- and antiapoptotic protein expressions [53].

### 6.3. Anticonvulsant Activity

The methanolic fraction of chloroform soluble part of the ethanolic extract of *A. lebbeck* (20, 40, or 100 mg/kg i.p.) exhibited remarkable anticonvulsant activity against pentylenetetrazole-induced convulsions and maximum electroshock in mice by delaying the onset of spasms and clonic convulsions. Fraction also delayed the latency to stage 4 significantly in lithium-pilocarpine-induced seizures. Moreover, in electrical kindling, fractions decreased the behavioral score. However, the fraction showed no protective effect against strychnine-induced convulsions [54]. Furthermore, 200 and 400 mg/kg (p.o.) ethanolic extract of *A. lebbeck* leaves demonstrated a considerable anticonvulsant effect by reducing the duration of hind limb extensor in the MES model and delaying the onset of convulsions in the PTZ mode [55].

### 6.4. Antidiabetic Activity

The bark of *A. lebbeck* demonstrated noteworthy antidiabetic activity. The methanol extract (200, 350, and 620 mg/kg) exhibited antihyperglycemic activity against streptozotocin-nicotinamide stimulated type II diabetes mellitus rats by significantly decreasing the level of serum glucose, creatinine, urea, cholesterol, triglycerides, LDL-cholesterol, and VLDL-cholesterol and increasing HDL levels as compared to diabetic control [16]. A study was conducted to evaluate *in vitro* antidiabetic activity of geraldone, isookanin, and luteolin isolated from methanolic extract of *A. lebbeck* bark, which showed potent inhibition against *α*-glucosidase and *α*-amylase (73.14 to 93.98%). The mechanistic approach of geraldone, isookanin, and luteolin has been graphically represented in Figure 7. In another study, it was demonstrated that methanol/dichloromethane extract of *A. lebbeck* bark possesses antidiabetic activity in streptozotocin-induced diabetic rats via significant reduction of blood glucose, BUN, SCr, GSP, TC, TG, LDL-c, and VLDL-c and increases plasma insulin level, hepatic enzymes, SOD, CAT, GSH, and HDL-c levels [17,18].

### 6.5. Anti-Inflammatory Activity

Administration of leaf essential oil (100, 200, and 400 mg/kg) caused significant inhibition of carrageenan-induced edema [11]. Leaves aqueous and ethanolic extract showed anti-inflammatory effect at 200 mg/kg with percentage inhibition of 39.36% and 42.55% in carrageenan-induced paw edema and also reduced granuloma formation with 38.55% and 42.33%, respectively [49]. In another study, petroleum ether and ethanol extracts (400 mg/kg) exhibited maximum inhibition of carrageenan-induced inflammation with percentage inhibition of 48.6% and 59.57%; dextran-induced group 45.99% and 52.93%; cotton pellet-induced models 34.46% and 53.57%, and Freund's adjuvant-induced animal group 64.97% and 68.57%, respectively [28], while bark petroleum ether: ethyl acetate: methanol extract (1 : 1 : 1) significantly (*p* < 0.001) reduces carrageenan-induced rat hind paw edema at 400 mg/kg with 36.68% [56]. Moreover, n-hexane, dichloromethane, ethyl acetate, and n-butanol fraction from flowers reduce inflammation in carrageenan-induced paw edema. Among tested fractions, the most potent activity was shown at 1 g/kg by dichloromethane (71.6%) followed by ethyl acetate (60.3%) [37].

### 6.6. Antimicrobial Activity

The zinc nanoparticle from the stem bark of *A. lebbeck* demonstrated activity against *B. cereus*, *S. aureus*, *E. coli*, *K. pneumoniae*, and *S. typhi*, with inhibition zones ranging from 1 to 10.57 mm, with *S. typhi* showing maximum inhibition at 0.1 M, which was comparable to ciprofloxacin (12.53 mm) [57]. In another study, ethanolic extract of root exerted antibacterial activity against *E. coli*, *S. flexneri*, *P. aeruginosa*, *S. typhi*, *K. pneumonia*, *S. boydii*, *S. aureus*, and *E. faecalis* with 9.05–15.77 mm inhibition range, where *S. typhi* showed maximum inhibition followed by *S. flexneri* (15.50 mm) at 200 mg/ml with MIC 0.20 and 0.39 mg/ml, respectively [30]. Similarly, petroleum ether, ethyl acetate, and methanol extracts from the stem bark and leaves exhibited antimicrobial activity against selective microbes among Gram-positive bacteria, that is, *B. polymyxa*, *B. subtilis*, *B. megaterium*, *S. lutea*, and *S. aureus*; Gram-negative bacteria such as *V. mimicus*, *V. cholera*, *S. typhi*, *S. boydii*, *S. flexneri* type-1, *S. dysenteriae*, *P. aeruginosa*, *K. pneumoniae*, *E. coli*, and *P. vulgaris*; fungal strains as *C. arrizae*, *A. fumigatus*, *A. Niger*, *R. oryzae*, *C. albicans*, *C. krusei*, and *Saccharomyces cerevisiae*. Stem bark extract was shown to have action with a zone of inhibition of 6–14 mm, with ethyl acetate extract having the best activity against *B. subtilis*, *S. typhi* (14 mm), and *C. arrizae* (10 mm). However, leaves extract had an antimicrobial activity with a zone of inhibition of 3–23 mm, whereas methanolic extract demonstrated the highest effective action against *S. typhi* at 500 mg [19, 58]. Moreover, leaves crude ethanolic extract at 10 mg/ml exerted activity against *S. aureus* (6 mm) and *E. coli* (7.5 mm), with IC_50_ 7.97, 5.62 mg/ml [52].

### 6.7. Antinociceptive Activity

Essential oil isolated from leaves significantly inhibited nociceptive mediators at both neurogenic and inflammatory phases in the formalin hind paw with an average of 44% and 100% at 200 and 400 mg/kg, respectively [11]. Leaves aqueous and ethanolic extract was administered orally to evaluate analgesic activity by eddy's hot plate and tail-flick test. In the hot plate method, a significant elevation was observed in the mean basal reaction time, and an elevation in the latency time was found in the tail-flick method [26]. In another study, among n-hexane, dichloromethane, ethyl acetate, and n-butanol fraction from flower, only dichloromethane fraction (1 g/kg) significantly increases in pain threshold in the hot plate test [37]. Bark petroleum ether: ethyl acetate: methanol extract (1 : 1 : 1) showed a significant reduction in the number of writhes by 52.4% and significant elongation of tail flicking time with 61.48% at 400 mg/kg [56].

### 6.8. Antioxidant Activity

Increased production of reactive oxygen species is a cause of most human diseases, including cardiovascular disease and cancer. Cells enable upregulation of antioxidant defenses and other protective systems against mild oxidative stress, although severe stress can harm the integrity of DNA, proteins, and lipids and lead to cell death by apoptotic or necrotic mechanisms [59]. Therefore, the antioxidant effect of *A. lebbeck* is evaluated. Geraldone, isookanin, and luteolin isolated from the bark of the plant are tested for DPPH-free radical scavenging assay, where geraldone showed the best activity (IC_50_ 21.5 *μ*M) [17]. These isolated compounds are able to neutralize the free radicals, including RNS and ROS, by activating antioxidant enzymes (Figure 7). Zinc oxide nanoparticles from the stem bark exhibited the most potent antioxidant effect against hydrogen peroxide-free radical with IC_50_ 48.5 *μ*g/ml [57]. Petroleum ether, ethyl acetate, and methanol barks extracts of *A. lebbeck* were evaluated for DPPH-free radical scavenging activity, where ethyl acetate (81.13%) and methanol extract (78.23%) showed high radical scavenging activity, followed by petroleum ether (74.82%) at 100 *μ*g/ml [58]. Additionally, leaves crude methanol extract showed DPPH and ABTS radical scavenging activity with IC_50_ 34.22 and 108.7 *μ*g/ml, respectively [52].

### 6.9. Antiparasitic Activity

Ethanolic extract from the bark of *A. lebbeck* showed antimalarial activity against *P. falciparum* chloroquine-resistant (RKL9) and CQ sensitive (MRC2) strains with IC_50_ 5.1 and 8.2 *μ*g/ml, respectively. Furthermore, the extract showed significant (*p* < 0.001) schizonticidal activity, repository, and curative activities against *P. berghei*. Moreover, plant extracts at different doses, 100, 250, 500, 750, and 1000 mg/kg/day, exhibited chemosuppression of 69.4, 71.4, 71.9, 79.8, and 84.7%, respectively, on the seventh day of postexposure [14]. In another study, pericarp ethanolic extract exhibited antiparasitic activity against *P. falciparum*, *L. infantum*, *T. cruzi*, and *T. brucei* with IC_50_ 37.9, 50.8, 8.7, and 8.1 *μ*g/ml, respectively [60].

### 6.10. Antivenom Activity

*Albizia lebbeck* is used traditionally as medicine in the treatment of snakebite, and several researchers have experimentally evaluated the medicinal use of *A. lebbeck* against snakebite [9, 10, 31]. One of the studies revealed that seed methanolic extract exhibited significant (*p* < 0.0001) antivenom activity with inhibition of ECV protease and hyaluronidase with IC_50_ 36.32 *μ*g, 91.95 *μ*g at 1 : 100 w/w, respectively. Moreover, extract neutralizes (*p* < 0.0001) ECV-induced hemorrhage with ED_50_ 26.37 *μ*g, myotoxicity by reducing serum creatinine kinase with ED_50_ 37.5 *μ*g (*p* < 0.0001), and lactate dehydrogenase 31.44 *μ*g (*p* = 0.0021) levels at 1 : 50 w/w [10].

### 6.11. Neuroprotective Activity

The symptoms of Alzheimer's disease include deterioration of memory, judgment, and decision-making power which reduces impairment in the orientation of physical surroundings and language [61]. It was observed that seed hydromethanolic extract (100–300 mg/kg orally) reduced biochemical oxidative stress and improved functional outcomes of behavioral studies by improving memory and cognition functions *via* inhibiting anticholinesterase, thereby preserving acetylcholine concentration [21]. The second most common neurodegenerative disease is Parkinson's disease which causes parkinsonism that occurs due to the loss of neurons in the substantia nigra and elsewhere in association with the presence of ubiquitinated protein deposits in the cytoplasm of neurons and thread-like proteinaceous inclusions within neurites [61]. The anti-Parkinson activity was evaluated by performing behavioral and biochemical oxidative stress assay in Wistar albino rats. It was observed that the plant extract can be able to ameliorate motor function and prevent biochemical damage in brain cells [12].

### 6.12. Nootropic Activity

The n-butanol fraction (10 and 25 mg/kg) from dried leaves of *A. lebbeck* exhibited excellent nootropic activity in mice by using the elevated plus maze and passive shock avoidance paradigm. On both doses, the inflexion ratio (IR) was increased significantly, while IR was found to decrease at the utmost dose (50 mg/kg) after 24 h after exposure as well as on day 9 in the passive avoidance test. Moreover, the fraction (10, 25, and 50 mg/kg) dose-dependently reduced the lithium-induced head twitches and at 50 mg/kg significantly potentiated and prolonged the haloperidol-induced catalepsy [20].

### 6.13. Miscellaneous Activity

Ovicidal and adulticidal activities were studied against *Culex quinquefasciatus*, *Aedes aegypti*, and *Anopheles stephensi* from hexane, benzene, chloroform, ethyl acetate, and methanol extracts; among tested extracts, methanolic extract obtained from the leaf and seed showed absolute mortality at 200, 250, 150, and 300, 375, and 225 ppm against *Ae. aegypti*, *C. quinquefasciatus*, and *An. stephensi*, respectively. Methanol leaf extract showed the highest adulticidal activity against *An. stephensi* with LC_50_ 65.12 ppm [62]. n-Hexane, dichloromethane, ethyl acetate, and n-butanol fraction from flower were evaluated for antipyretic activity. The most potent effect was shown by dichloromethane followed by ethyl acetate at 1 g/kg with a reduction of 8°C and 5°C, respectively [37]. Aqueous methanol extract from seed (5 mg/kg *i.p.*) almost entirely inhibits the castor oil-induced diarrhea [63]. The pharmacological profile of various parts of *A. lebbeck* is shown in Table 4.

## 7. Conclusion

*Albizia lebbeck* is an Ayurvedic plant and has been widely utilized in the treatment of anorectal, eye, gastrointestinal, genital, inflammatory, neurological disorders, oral disorders, respiratory, skin, urinary disorders, and venereal diseases across the world. Different parts of the plant have been used, but bark appears to be the most often used plant part in the employment of traditional medicine. However, in support of its therapeutic uses, more scientific clinical trials extensively are necessary. The phytochemical studies revealed an abundance of saponins with other chemicals, for example, flavonoids, phenols, and glycosides. *A. lebbeck* has been studied for many pharmacological activities against allergy, cancer, convulsant, diabetes, inflammation, parasitic infestation, snake venom, nootropic, pyrexia, diarrhea, and so on, and there remains still a scarcity of information on the mechanism of action. Additionally, it is worth noting that even though *A. lebbeck* has been used in the treatment of various ailments, it is an ingredient in several Ayurvedic formulations; nonetheless, studies are required to evaluate the possible toxicities or adverse effects. In forthcoming research, studies should target the discovery of the chemical compounds responsible for the therapeutic action, which comprise the mechanisms of action.

## Figures and Tables

**Table 1:** Ethnomedicinal uses of different parts of *A. lebbeck* in various traditional medicinal systems.

Parts used	Medicinal system	Mode of administration	Ethnomedicinal uses	References
Bark	Indian traditional medicine		Asthma, bronchitis, arthritis, gingivitis, toothache, allergies, leukoderma, leprosy, snakebites, malaria, and fractures	[15, 26]
Leaves			Night blindness and syphilis	[26]
All parts			Snakebite, scorpion sting, hemorrhage, and gum inflammation	[10]
Bark and flowers	Siddha system		Arthritis	[18]
Flowers	Traditional Chinese medicine		Anxiety, depression, and insomnia	[27]
	Ayurveda		Nasya, pittaja, prameha, asthma, arthritis, burns, diarrhea, edema, poisoning, bronchitis, consumption, night blindness, respiratory disorders, skin disorders, snakebite, and scorpion sting	[3, 4, 27–29]
Root			Wounds	[30]
Bark			Bronchitis, leprosy, paralysis, gum inflammation, and helminthic infection	[3]
Leaves		Poultice	Night blindness and ulcer	[3]
Flower		Juice	Poisoning, hikka (hiccup), shwasa (asthma), and eye disease	[16]
Seed			Piles and diarrhea	[5]

**Table 2:** Medicinal uses of *A. lebbeck* in different countries of the world.

S. no.	Country	Parts used	Mode of administration	Medicinal uses	References
1	Africa	Leaves, stem bark, pods, and seeds	—	Dysentery, diarrhea, bronchial asthma, eczema, insect bite, allergy, piles, hernia, malaria, gonorrhea, scrofulous swellings, earache, antiprotozoal, and anthelmintic	[7]
	Zulu of Southern Africa	Bark and roots	—	Scabies, inflamed eyes, piles, and bronchitis	[11]
	West Africa		—	Diarrhea, dysentery, hemorrhoids, bronchitis, asthma, eczema, and leprosy	[31, 38]
2	Asia	Stem	—	Abdominal tumors, boils, cough, eye disorders, and lung ailments	[31]
		Bark			
3	Australia	Seed, = stem bark, and root bark	—	Diarrhea, gastroenteritis, hemorrhoids, bronchitis, leprosy, paralysis, parasitic infestation, ulcer, snakebite, gum ailments, abdominal tumors, boils, cough, eye disorders, and lung ailments	[31, 39]
4	Bangladesh	Bark, seed, and leaves	Decoction	Piles, diarrhea, toothache, gum ailments, bronchial asthma, allergic disorder, and ophthalmia	[36]
5	China	Flowers		Anxiety, depression, and insomnia	[27]
6	India	Bark and seed	Powder and juice	Astringent, tonic, restorative, and anus pain	[40, 41]
		Bark, flowers, seeds, and roots		Arthritis, bone fracture, edema, poisoning, asthma, bronchitis, skin disease, cold and cough, itching, pruritus, wounds healing, leprosy, malaria, gonorrhea, abscesses, boils and abdominal tumors, snakebite, scorpion sting, hemorrhage, and gum inflammation	[9, 10]
				Spermatorrhea	[42]
	India (Bhils and Meena tribes)	Stem bark, flowers, and leaves	Powder, paste, and decoction	Stone, boil, pimples, ulcer, gums ailments, pharyngitis, insect bite, and scorpion sting	[8]
	India (tribes of Himachal Pradesh and Kashmir)			Inflammation	[28]
	India (Tamil Nadu)			Bone fractures	
7	Myanmar (Burma)			Abdominal tumors	[9]
8	Nepal	Root, leaves, flowers, bark, and seed	Bark aqueous extract (leaf), decoction (seed), ointment, and powder	Snakebite, scorpion sting, hemicrania, strengthen gum, ophthalmia, cough, bronchitis, asthma, prevent conception in women, anus pain, night blindness, astringent, piles, diarrhea, dysentery, gums ailment (spongy and ulcerated gums), emollient for boils, eruption, carbuncle, swelling, eye disease, and scrofulous enlargement of glands	[6, 40, 43]
9	Nigeria		Aqueous extract	Fever, pain, epilepsy, and inflammation	[11]
10	Philippines	Bark and leaves	Decoction	Dysentery, diarrhea, and ulcer	[44]
11	Taiwan	Bark		Anthelmintic, diuretic, stimulant, tonic, and vermifuge	[35]
12	Tibet			Kapha, pitta, poisoning, erysipelas, and ulcer	[34]

**Table 3:** Chemicals constituents of *A. lebbeck*.

Chemical compounds	Plant part	References
Alkaloids (budmunchiamines L1–L6), *α*-amyrine, catechins, echinocystic acid or acacic acid, flavonoids (kaempferol, quercetin, and quercetin 3-O-alpha-rhamnopyranosyl (1 ⟶ 6)-beta-glucopyranosyl (1 ⟶ 6)-beta-galactopyranosides), friedelan-3-one, (−)-leucopelargonidin, lupeol, melanoxetin, okanin, oleanolic acid, (+) pinitol, polyphenols, saponins (lebbekanin A-H) g-sitosterol, and triterpenoids	Plant	[15, 28, 48]
Oleanane-type saponins (lebbeckosides A and B)	Roots	[31]
Alkaloids, flavonoid (geraldone, luteolin, isookanin, epicatechin, and procyanidins B-2, B-5, and C-3), glycoside (albizinin), hemolysin (lebbeckalysin), oleanane triterpene (albiziasaponins A–E), phenols, phytosterols, saponins, and triterpenoid saponin (lebbeckoside C, 21-[[(2e,6S)-6-[6-deoxy-4-O-[(2e,6S)-6-hydroxy-2-(hydroxymethyl)-6-methyl-1-oxo-2,7-octadienyl]-[(*β*-D-glucopyranosyl) oxy]-2-(hydroxymethyl)-6-methyl-1-oxo-2,7-octadienyl]-[(*β*-D-gluco- pyranosyl)oxy]-2,6-dimethyl-1-oxo-2,7-octadienyl]oxy]-16-hydroxy-3-[[O-*β*-D-xylopyranosyl-(1 ⟶ 2)-O-*α*-L-arabinopyranosyl-(1 ⟶ 6)-2-(acet-ylamino)-2-deoxy-*β*-D-glucopyranosyl]oxy]-(3*β*,16*α*,21*β*)-olean-12-en-28-oic acid O-*α*-L-arabinofuranosyl-(1 ⟶ 4)-O-[*β*-D-glucopyranosyl- (1 ⟶ 3)]-O-6-deoxy-*α*-L-mannopyranosyl-(1 ⟶ 2)-*β*-D-glucopyranosyl ester)	Bark	[4, 12, 14, 17, 38, 46, 47]
Alkaloids, glycosides, saponin (albiziahexoside) steroids, tannins, terpenoids, flavonoids (kaempferol 3-O-a rhamnopyranosyl(1/6)-b-glucopyranosyl(1/6)-o-galactopyranoside, quercetin 3-O-a rhamnopyranosyl(1/6)-b-glucopyranosyl(1/6)-b-galactopyranoside, kaempferol, and 3-rhamnosyl (1–6) glycosyl (1–6) galactoside)	Leaves	[4,49,50]
Alkaloids, anthraquinones, eicosane, fatty acid (linolenic acid, oleic acid, palmitic acid, and steric acid), flavonoids, glycosides, nonadecane, octadecane, phenolics, phytol, saponins (glycosaponins), steroids, stigmastadiene, tetradecane, and vitamin E	Seed	[10,21,45]
3′,5-Dihydroxy-4′,7 dimethoxy flavone and N-benzoyl-L-phenyl alaninol	Pod	[19]
Albigenic acid	Bean	

**Table 4:** Pharmacological activities of various parts of *A. lebbeck*.

S. no.	Pharmacological activity	Extract, fraction, and isolate	Parts used	Dose/mode of administration	Standard	Study model/parameter	Result	Ref.
1	Antiallergic activity	Ethanolic extract	Stem bark	50 to 300 mg/kg, p.o.	DSCG (50 mg/kg, i.p.)	Mast cell stabilization, compound 48/80-induced systemic anaphylaxis	Dose-dependent mast cell stabilization activity at 200 and 300 mg/kg dose extract protected the degranulation (53 and 61%, resp.). There was significant protection from degranulation (compound 48/80 induced) of mast cells, dose-dependent, that is, 61 and 74% of inhibition of histamine release at 200 and 300 mg/kg, respectively	[15]
		Chloroform, methanol, and water extracts	Leaf and stem bark	50 *μ*g/ml	1% DMSO	*In vitro* mesenteric mast cell stabilization against compound 48/80	All the extracts showed significant mast cell stabilization activity. However, methanolic and water extracts of the bark showed the maximum activity along with the leaf methanolic extract	[51]

2	Anti-Alzheimer's activity	Hydromethanolic extract	Seed	100–300 mg/kg p.o.	Galantamine 0.5 mg/kg	*In vivo* aluminum chloride (100 mg/kg, p.o.)-induced Alzheimer's disease in Wistar albino rats	Extracts significantly improved the memory and cognitive impairments, ↑GSH, SOD, CAT, and ↓ AChE	[21]
						Morris water maze, open field, hole board, Y-maze, and T-maze test		
3	Anticancer activity	Saponin-rich fraction	Bark	0.001, 0.01, 0.1, 1, and 10 *μ*g/ml	Doxorubicin 500 nM	*In vitro* MTT assay in human breast cancer MCF-7	Fraction inhibits the growth of MCF-7 with IC_50_ 1 *μ*g/ml	[9]
				10 *μ*g/ml	Staurosporine 1 ug/ml	Apoptosis assay	Fraction increases apoptosis and promotes activation of caspases 3 and 8	
				0.1, 0.5, and 1 *μ*g/ml		Shell-less chick embryo culture assay	Reduction in number of extremities, nodes, junctions, and total branches length between 0 and 3 hr and 0 and6 hr of drug exposure	
						Chromosomal aberration (CA) assay	↑ Total chromosomal aberrations	
		Zinc oxide nanoparticles	Stem bark	5, 25, 50, and 100 *μ*g/ml of 0.1 M, 0.05 M, and 0.01 M ZnO NPs		*In vitro* MTT assay in human breast cancer MCF-7 and MDA-MB 231	Extract significantly inhibited the viability	[57]
		Lebbeckosides A-B	Root		Tamoxifen	*In vitro* cytotoxicity against the glioblastoma stem-like TG1 cells and human glioblastoma U-87 MG cell lines	Lebbeckoside A and B showed cytotoxicity against TG1 and U-87 MG, with IC_50_ 2.10, 2.24, 3.46, and 1.36 *μ*M, respectively	[31]
		Crude methanol extract	Leaves	1, 10, 25, 50, 75, 100, 125, and 150 *μ*g/ml		*In vitro* MTT assay against human hepatocarcinoma (HepG2) cancer cell line	Extract significantly decreased the cell viability with IC_50_ 24.03 *μ*g/ml	[52]

4	Antidiabetic activity	Methanolic extract	Bark	200, 350, and 620 mg/kg/day, p.o.	Metformin 45 mg/kg	Streptozotocin-nicotinamide-induced type II diabetes mellitus using female Sprague-Dawley rats	Extract significantly decreased the level of serum GLU, creatinine, urea, triglycerides, cholesterol, low-density lipoprotein-cholesterol, and very low-density lipoprotein-cholesterol and increased high-density lipoprotein levels	[16]
		Geraldone, isookanin, and luteolin	Bark		Acarbose 10 mg/ml	*In vitro α*-glucosidase and *α*-amylase inhibitory assay	All three compounds significantly inhibit the *α*-glucosidase and *α*-amylase enzymes	[17]
		Methanol/dichloromethane extract	Stem bark	100–400 mg/kg	Glibenclamide 1 mg/kg	*In vivo* streptozotocin-induced diabetic rats using male albino Wistar rats	Significant reduction of blood glucose, BUN, SCr, GSP, TC, TG, LDL-c, and VLDL-c and increasing plasma insulin level, hepatic enzymes, SOD, CAT, GSH, and HDL-c	[18]

5	Antidiarrheal activity	Aqueous methanol extract	Seed	2.5–5 mg/kg i.p.	Loperamide 1 mg/kg i.p.	*In vivo* castor oil-induced diarrhea using albino rats and mice	Extract significantly inhibited the cathartic effect of castor oil in a dose-dependent manner	[63]
6	Anti-inflammatory activity	Essential oil	Leaves	100–400 mg/kg p.o.	Ibuprofen 100 mg/kg	*In vivo* carrageenan-induced edema in Wistar rats	Extract significantly and dose-dependently inhibited edema	[63]
		Aqueous and ethanolic extract	Leaves	50–200 mg/kg, p.o.	Diclofenac 20 mg/kg and indomethacin 10 mg/kg	*In vivo* carrageenan-induced paw edema and cotton pellet-induced granuloma models using Wistar rats	Dose-dependent and significant inhibition of inflammation	[49]
		Petroleum ether, chloroform, and ethanol extract	Bark	100, 200, and 400 mg/kg p.o.	Indomethacin 10 mg/kg	*In vivo* carrageenan- and dextran-induced rat paw edema; cotton pellet-induced granuloma; adjuvant-induced arthritis using female Wistar rats	Dose-dependent and significant inhibition of inflammation	[28]
		n-Hexane, dichloromethane, ethyl acetate, and n-butanol fraction	Flower	0.25 and 1 g/kg, i.p.	Diclofenac sodium 20 mg/kg	*In vivo* carrageenan-induced paw edema using Wistar rats	All fractions showed significant inhibition	[37]
		Petroleum ether: ethyl acetate: methanol extract (1 : 1:1)	Bark	200 and 400 mg/kg p.o.	Phenylbutazone 100 mg/kg	*In vivo* carrageenan-induced rat hind paw edema using long-Evans rats	Dose-dependent and significant inhibition of inflammation	[56]

7	Antimicrobial activity	Zinc oxide nanoparticles	Stem bark	0.01 M, 0.05 M, and 0.1 M	Ciprofloxacin 10 *μ*g/disc	*In vitro* agar disc diffusion method using *Bacillus cereus, Staphylococcus aureus, Escherichia coli, Klebsiella pneumoniae,* and *Salmonella typhi*	Extract showed strong activity with inhibition zone ranging from 1 to 10.57 mm	[57]
		Ethanolic extract	Root	100–200 mg/ml	Ciprofloxacin	*In vitro* disc diffusion method using *Escherichia coli, Shigella flexneri, Pseudomonas aeruginosa, Staphylococcus aureus*, and four clinical bacterial isolates *Salmonella typhi, Klebsiella pneumoniae, Shigella boydii*, and *Enterococcus faecalis*	Extract showed activity against all tested bacteria with a zone of inhibition ranging from 9.05 to 15.77 mm and MIC 0.20–1.56 mg/ml	[30]
		Petroleum ether, ethyl acetate, and methanol extracts	Stem bark	300 *μ*g/disc	Ciprofloxacin 10 *μ*g/disc for bacteria; griseofulvin 25 *μ*g/disc for fungi	*In vitro* disc diffusion method using *Bacillus polymyxa, B. subtilis, B. megaterium, Sarcina lutea, Staphylococcus aureus, Vibrio mimicus, V. Cholera, Salmonella typhi, Shigella boydii, S. flexneri* type-1*, S. dysenteriae, Pseudomonas aeruginosa, Klebsiella pneumoniae*, *Escherichia coli, Candida arrizae, Aspergillus fumigatus, A. niger*, *Rhizopus oryzae*, *Candida albicans, C. krusei,* and *Saccharomyces cerevisiae*	Pet. ether and ethyl acetate extract showed activity against selective microbes with ZOI ranging from 6 to 14 mm. Methanol extract is only active against *S. cerevisiae* (8 mm)	[58]
		Petroleum ether, ethyl acetate, and methanol extract	Leaves	50, 100, 200, and 500 *μ*g/ml	Tetracycline, streptomycin, erythromycin, lincomycin, rifampicin, norfloxacin, and gentamycin	*In vitro* agar disc diffusion method using *Bacillus subtilis, Escherichia coli, Klebsiella pneumonia, Proteus vulgaris, Pseudomonas aeruginosa, Salmonella typhi*, and *Staphylococcus aureus*	Among extracts, methanolic extract showed strong activity with a zone of inhibition ranging from 11 to 23 mm at 500 *μ*g/ml	[19]
		Crude methanol extract	Leaves	10 mg/ml	Ampicillin 10 mg/ml, streptomycin 10 mg/ml, and tetracycline 20 mg/ml	*In vitro* agar well diffusion method against *Staphylococcus aureus, Pseudomonas aeruginosa, Candida albicans,* and *Escherichia coli*	Extract showed potent antibacterial activity against *S. aureus* and *E. coli* with ZOI 6 and 7.5 mm, respectively	[52]
8	Antinociceptive activity	Essential oil	Leaves	100–400 mg/kg p.o.	Piroxicam 10 mg/kg p.o.	*In vivo* formalin hind paw in Wistar rats	Extract inhibited nociceptive mediators at both neurogenic and inflammatory phases	[11]
		Aqueous and ethanolic extract	Leaves	50–200 mg/kg, p.o.	Pentazocine 15 mg/kg	*In vivo* Eddy's hot plate and tail-flick test in Wistar rats	Both extracts showed a significant and dose-dependent increase in the mean basal reaction time in the hot plate test and latency of the flick tail response	[26]
		n-Hexane, dichloromethane, ethyl acetate, and n-butanol fraction	Flower	0.25 and 1 g/kg, i.p.	Aspirin 200 mg/kg	*In vivo* hot plate method using male albino white mice	Only dichloromethane fraction (1 g/kg) significantly increases in pain threshold	[37]
		Petroleum ether: ethyl acetate: methanol extract (1 : 1:1)	Bark	200 and 400 mg/kg p.o.	Aminopyrine 50 mg/kg	Acetic acid induced writhing test using Swiss albino mice	Extract showed a significant and dose-dependent reduction in the number of writhes	[56]
					Morphine 2 mg/kg	Radiant heat tail-flick method using Swiss albino mice	Extract showed significant elongation of tail flicking time	

9	Antioxidant activity	Zinc oxide nanoparticles	Stem bark	0.01, 0.05, and 0.1 M	Ascorbic acid	H_2_O_2_-free radical scavenging assay	IC_50_ 48.7, 60.2, and 48.5 *μ*g/ml, respectively	[57]
		Geraldone, isookanin, and luteolin	Bark		Trolox	DPPH radical scavenging assay	All compounds showed activity with IC_50_ 21.5, 31.8, and 29.26 *μ*M, respectively	[17]
		Petroleum ether, ethyl acetate, and methanol extracts	Stem bark	20–100 *μ*g/ml	Ascorbic acid	DPPH- and H_2_O_2_-free radical scavenging assay	Extracts showed DPPH- and H_2_O_2_-free radical scavenging activity with IC_50_ values of 66.63, 57.25, 60.21, 70.93, 64.69, and 68.99 *μ*g/ml, respectively	[58]
		Crude methanol extract	Leaves	1, 10, 25, 50, 75, 100, 125, and 150 *μ*g/ml	Ascorbic acid	DPPH and ABTS radical scavenging assays	Extract exhibited DPPH and ABTS radical scavenging activity with IC_50_ 34.22 and 108.7 *μ*g/ml, respectively	[52]

10	Antiparasitic activity	Ethanolic extract	Bark	5–100 *μ*g/ml	Chloroquine 5 mg/kg	*In vitro* antimalarial activity against *Plasmodium falciparum* chloroquine (CQ) sensitive (MRC2) and CQ resistant (RKL9) strains	IC_50_ = 8.2 and 5.1 *μ*g/ml against MRC2 and RKL9 strains	[14]
		Ethanolic extract	Bark	100, 250, 500, 750, and 1000 mg/kg/day	Chloroquine 5 mg/kg and pyrimethamine 1.25 mg/kg	*In vivo* schizonticidal activity, repository, and curative activities using *P. berghei*-infected white Swiss albino mice	Dose-dependent chemosuppression was observed with significant schizonticidal activity at 1000 mg/kg with ED > 100 mg/kg. Significant curative and repository activities were exhibited by 750 mg/kg concentration of extract on day 7	
		Methanol extract	Pericarp	20 mg/ml	Chloroquine, miltefosine, benznidazole, and suramin	*In vitro* antiplasmodial, antileishmanial, and antitrypanosomal activities against *Plasmodium falciparum*, *Leishmania infantum*, *Trypanosoma cruzi*, and *T. brucei*	Extract showed antiparasitic activity with IC_50_ 8.7, 8.1, 37.9, and 50.8 *μ*g/ml against *T. cruzi*, *T. brucei*, *P. falciparum,* and *L. infantum,* respectively	[60]

11	Anti-Parkinson's activity	Aqueous methanolic extract	Seed	100–300 mg/kg	Sinemet-levodopa 100 mg + carbidopa 25 mg/kg per oral	*In vivo* haloperidol-induced catalepsy	Extract improved the motor functions and showed significant improvement in catalepsy, time latency, no. of exploration, ↑ SOD, CAT, and GSH	[12]
						Assessment of catalepsy, hang test, and narrow beam walk test		
						Open field test		
12	Antipyretic activity	n-Hexane, dichloromethane, ethyl acetate, and n-butanol fraction	Flower	0.25 and 1 g/kg, i.p.	Aspirin (200 mg/kg)	*In vivo* Brewer's yeast-induced pyrexia using albino mice	All fractions showed a decrease in temperature	[37]
13	Antivenom activity	Methanolic extract	Seed	1: 1–1: 100 w/w		*Echis carinatus* venom- (ECV-) induced local toxicity in Swiss albino mice *in vivo* and proteolytic and hyaluronidase activities *in vitro*	Extract inhibited protease and hyaluronidase (IC_50_ 36.32 and 91.95 *μ*g), hemorrhage (ED_50_ 26.37 *μ*g), serum creatinine kinase, and lactate dehydrogenase (ED_50_ 37.5 and 31.44 *μ*g)	[10]
14	Estrogenic activity	n-Hexane, dichloromethane, ethyl acetate, and n-butanol fraction	Flower	200 and 500 mg/kg i.p.	17-*β*-Estradiol (0.32 *μ*g/animal/day)	Uterine weight using female Albino mice	Ethyl acetate (200) and total alcohol fraction (500 mg/kg) significantly decrease and increase uterine weight by 25.2 and 109%, respectively	[37]
15	Wound healing activity	Ethanolic extract	Root	250, 500, and 750 mg/kg p.o.	Vitamin E 200 mg/kg	*In vivo* incision and excision wound models in nulliparous and nonpregnant healthy female rats	↑ Wound breaking strength in incision model, complete wound contraction was observed on the 22nd day in excision model, ↑ wet weight of granulation tissue, total protein, SOD, GSH, hydroxyproline, hexosamine, hexuronic acid levels, ↓ lipid peroxidation, and nitric oxide	[30]

## References

[1] He Y., Wang Q., Ye Y., Liu Z., Sun H. (2020). The ethnopharmacology, phytochemistry, pharmacology and toxicology of genus *Albizia*: a review. *Journal of Ethnopharmacology*.

[2] Parrotta J. A. (2006). *Albizia lebbek* [monograph]. *“Enzyklopädie der Holzgewächse”[Encyclopaedia of Woody Plants]*.

[3] Kapoor L. D. (2001). *Handbook of Ayurvedic Medicinal Plants*.

[4] Sarin Y. K. (1996). *Illustrated Manual of Herbal Drugs Used in Ayurveda*.

[5] Drury C. H. (2010). *Ayurvedic Useful Plants of India: With Their Medicinal Properties and Uses in Commerce, Medicine and Arts*.

[6] Baral S. R., Kurmi P. P. (2006). *A Compendium of Medicinal Plants in Nepal*.

[7] Iwu M. M. (2014). *Handbook of African Medicinal Plants*.

[8] Singh V., Pandey R. P. *Ethnobotany of Rajasthan*.

[9] Desai T. H., Joshi S. V. (2019). Anticancer activity of saponin isolated from *Albizia lebbeck* using various in vitro models. *Journal of Ethnopharmacology*.

[10] Amog P. U., Manjuprasanna V. N., Yariswamy M. (2016). Albizia lebbeckseed methanolic extract as a complementary therapy to manage local toxicity ofEchis carinatusvenom in a murine model. *Pharmaceutical Biology*.

[11] Avoseh O. N., Mtunzi F. M., Ogunwande I. A., Ascrizzi R., Guido F. (2021). *Albizia lebbeck* and *Albizia zygia* volatile oils exhibit anti-nociceptive and anti-inflammatory properties in pain models. *Journal of Ethnopharmacology*.

[12] Saleem U., Raza Z., Anwar F., Chaudary Z., Ahmad B. (2019). Systems pharmacology based approach to investigate the *in-vivo* therapeutic efficacy of *Albizia lebbeck* (L.) in experimental model of Parkinson’s disease. *BMC Complementary and Alternative Medicine*.

[13] Ahmed S., Hassan B., Saleem M. U., Riaz M. A., Nisar M. S. (2018). Efficacy of heartwood extractives of *Albizia lebbeck* (L.) Benth. against subterranean termites. *International Wood Products Journal*.

[14] Kalia S., Walter N. S., Bagai U. (2015). Antimalarial efficacy of *Albizia lebbeck* (Leguminosae) against *Plasmodium falciparumin vitro* & *P. berghei in vivo*. *Indian Journal of Medical Research*.

[15] Venkatesh P., Mukherjee P. K., Kumar N. S. (2010). Anti-allergic activity of standardized extract ofAlbizia lebbeckwith reference to catechin as a phytomarker. *Immunopharmacology and Immunotoxicology*.

[16] Patel P. A., Parikh M. P., Johari S., Gandhi T. R. (2015). Antihyperglycemic activity of *Albizzia lebbeck* bark extract in streptozotocin-nicotinamide induced type II diabetes mellitus rats. *Ayu*.

[17] Ahmed D., Kumar V., Sharma M., Verma A. (2014a). Target guided isolation, *in-vitro* antidiabetic, antioxidant activity and molecular docking studies of some flavonoids from *Albizzia Lebbeck* Benth. bark. *BMC Complementary and Alternative Medicine*.

[18] Ahmed D., Kumar V., Verma A. (2014b). Antidiabetic, renal/hepatic/pancreas/cardiac protective and antioxidant potential of methanol/dichloromethane extract of *Albizzia lebbeck* Benth. stem bark (ALEx) on streptozotocin induced diabetic rats. *BMC Complementary and Alternative Medicine*.

[19] Bobby M. N., Wesely E. G., Johnson M. A. (2012). *In vitro* anti- bacterial activity of leaves extracts of *Albizia lebbeck* Benth against some selected pathogens. *Asian Pacific Journal of Tropical Biomedicine*.

[20] Chintawar S. D., Somani R. S., Kasture V. S., Kasture S. B. (2002). Nootropic activity of *Albizzia lebbeck* in mice. *Journal of Ethnopharmacology*.

[21] Saleem U., Raza Z., Anwar F., Ahmad B., Hira S., Ali T. (2019). Experimental and computational studies to characterize and evaluate the therapeutic effect of Albizia lebbeck (L.) seeds in Alzheimer’s disease. *Medicina*.

[22] Kavaz D., Abubakar A. L., Rizaner N., Umar H. (2021). Biosynthesized ZnO nanoparticles using *Albizia lebbeck* extract induced biochemical and morphological alterations in wistar rats. *Molecules*.

[23] Waseem A., Jamal A., Ahmad W., Fazil M. (2020). Siras (*Albizia lebbeck* (L.) Benth.) and its medicinal uses in Unani medicine-a review. *Cell Medicine*.

[24] Shirisha K., Priyanka B., Rahman H., Bardalai D., Ali F. (2013). Review on *Albizia lebbeck* (L.) Benth: a plant possessing diverse pharmacological activities. *Research Journal of Pharmacognosy and Phytochemistry*.

[25] Verma S. C., Vashishth E., Singh R. (2013). A review on parts of *Albizia lebbeck* (L.) Benth. used as ayurvedic drugs. *Research Journal of Pharmacy and Technology*.

[26] Meshram G. G., Kumar A., Rizvi W., Tripathi C. D., Khan R. A. (2015). Central analgesic activity of the aqueous and ethanolic extracts of the leaves of *Albizia lebbeck*: role of the GABAergic and serotonergic pathways. *Zeitschrift für Naturforschung C*.

[27] Al-Massarani S. M., El Gamal A. A., Abd El Halim M. F. (2017). New acyclic secondary metabolites from the biologically active fraction of *Albizia lebbeck* flowers. *Saudi Pharmaceutical Journal*.

[28] Babu N. P., Pandikumar P., Ignacimuthu S. (2009). Anti-inflammatory activity of *Albizia lebbeck* Benth., an ethnomedicinal plant, in acute and chronic animal models of inflammation. *Journal of Ethnopharmacology*.

[29] Mishra L. C. (2004). *Scientific Basis for Ayurvedic Therapies*.

[30] Joshi A., Sengar N., Prasad S., Goel R., Singh A., Hemalatha S. (2013). Wound-healing potential of the root extract of *Albizzia lebbeck*. *Planta Medica*.

[31] Noté O. P., Jihu D., Antheaume C. (2015). Triterpenoid saponins from *Albizia lebbeck* (L.) Benth and their inhibitory effect on the survival of high grade human brain tumor cells. *Carbohydrate Research*.

[32] (2016). *The Ayurvedic Pharmacopoeia of India, part 1*.

[33] Rahman A., Choudhary M. I., Bullo S. (2006). *Monograph Medicinal Plants of Sindh: Indigenous Knowledge and Scientific Facts*.

[34] Dash B. (1994). *Materia Medica of Tibetan Medicine*.

[35] Li T. S. C. (2006). *Taiwanese Native Medicinal Plants*.

[36] Ghani A. (2003). *Medicinal Plants of Bangladesh with Chemical Constituents and Uses*.

[37] Farag M., Gamal A. E., Kalil A., Al-Rehaily A., Mirghany O. E., Tahir K. E. (2013). Evaluation of Some Biological Activities of &lt;i&gt;Albizia lebbeck&lt;/i&gt; Flowers. *Pharmacology &amp; Pharmacy*.

[38] Noté O. P., Ngo Mbing J., Kilhoffer M. C., Pegnyemb D. E., Lobstein A. (2019). Lebbeckoside C, a new triterpenoid saponin from the stem barks of *Albizia lebbeck* inhibits the growth of human glioblastoma cells. *Natural Product Research*.

[39] Williams C. (2011). Medicinal plants in Australia. *Gums, Resins, Tannin*.

[40] Singh V. (2009). *Ethnobotany and Medicinal Plants of India and Nepal*.

[41] Trivedi P. C. (2009). *Medicinal Plants: Utilization and Conservation*.

[42] Gupta R. S., Chaudhary R., Yadav R. K., Verma S. K., Dobhal M. P. (2005). Effect of Saponins of *Albizia lebbeck* (L.) Benth bark on the reproductive system of male albino rats. *Journal of Ethnopharmacology*.

[43] Kayastha B. P. (2002). *A Handbook of Trees of Nepal*.

[44] Batugal P. A., Kanniah J., Sy L., Oliver J. T. (2004). Medicinal plants research in Asia. *The Framework and Project Workplans*.

[45] Adewuyi A., Oderinde R. A. (2014). Fatty acid composition and lipid profile of *Diospyrosme spiliformis, Albizia lebbeck*, and *Caesalpinia pulcherrima* seed oils from Nigeria. *International Journal of Food Science*.

[46] Viana E. O. R., Cruz M. d. F. S. J., da Silva M. J. (2019). Structural characterization of a complex triterpenoid saponin from *Albizia lebbeck* and investigation of its permeability property and supramolecular interactions with membrane constituents. *Carbohydrate Research*.

[47] Lam S. K., Ng T. B. (2011). First report of an anti-tumor, anti-fungal, anti-yeast and anti-bacterial hemolysin from *Albizia lebbeck* seeds. *Phytomedicine*.

[48] Nurul I. M., Mizuguchi H., Shahriar M. (2011). Albizia lebbeck suppresses histamine signaling by the inhibition of histamine H1 receptor and histidine decarboxylase gene transcriptions. *International Immunopharmacology*.

[49] Meshram G. G., Kumar A., Rizvi W., Tripathi C. D., Khan R. A. (2016). Evaluation of the anti-inflammatory activity of the aqueous and ethanolic extracts of the leaves of *Albizzia lebbeck* in rats. *Journal of Traditional and Complementary Medicine*.

[50] Ueda M., Tokunaga T., Okazaki M., Sata N. U., Ueda K., Yamamura S. (2003). Albiziahexoside: a potential source of bioactive saponin from the leaves ofAlbizzia lebbeck. *Natural Product Research*.

[51] Shashidhara S., Bhandarkar A. V., Deepak M. (2008). Comparative evaluation of successive extracts of leaf and stem bark of *Albizzia lebbeck* for mast cell stabilization activity. *Fitoterapia*.

[52] Singh G., Passsari A. K., Leo V. V. (2016). Evaluation of phenolic content variability along with antioxidant, antimicrobial, and cytotoxic potential of selected traditional medicinal plants from India. *Frontiers of Plant Science*.

[53] Malaikolundhan H., Mookkan G., Krishnamoorthi G. (2020). Anticarcinogenic effect of gold nanoparticles synthesized from *Albizia lebbeck* on HCT-116 colon cancer cell lines. *Artificial Cells, Nanomedicine, and Biotechnology*.

[54] Kasture V. S., Chopde C. T., Deshmukh V. K. (2000). Anticonvulsive activity of Albizzia lebbeck, Hibiscus rosa sinesis and Butea monosperma in experimental animals. *Journal of Ethnopharmacology*.

[55] Srivastav Neeti S. S., Vijay J., Tiwari Brijesh K. (2016). Anti-convulsant activity of leaf extracts of *Albizia lebbeck* linnn in experimental rats. *International Journal of Pharmaceutical Sciences Review and Research*.

[56] Saha A., Ahmed M. (2009). The analgesic and anti-inflammatory activities of the extract of *Albizia lebbeck* in animal model. *Pakistan Journal of Pharmaceutical Sciences*.

[57] Umar H., Kavaz D., Rizaner N. (2019). Biosynthesis of zinc oxide nanoparticles using *Albizia lebbeck* stem bark, and evaluation of its antimicrobial, antioxidant, and cytotoxic activities on human breast cancer cell lines. *International Journal of Nanomedicine*.

[58] Ali M. T., Haque S. T., Kabir M. L., Rana S., Haque M. E. (2018). A comparative study of in vitro antimicrobial, antioxidant and cytotoxic activity of *Albizia lebbeck* and *Acacia nilotica* stem bark. *Bulletin of the Faculty of Pharmacy Cairo University*.

[59] Priyanka B., Anitha K., Shirisha K., Sk J., Dipankar B., Rajesh K. (2013). Evaluation of anti-oxidant activity of ethanolic root extract of *Albizia lebbeck* (l.) Benth. *International Research Journal of Pharmaceutical and Applied Sciences*.

[60] Al-Musayeib N. M., Mothana R. A., Al-Massarani S., Matheeussen A., Cos P., Maes L. (2012). Study of the *in vitro* antiplasmodial, antileishmanial and antitrypanosomal activities of medicinal plants from Saudi Arabia. *Molecules*.

[61] Nussbaum R. L., Ellis C. E. (2003). Alzheimer’s disease and Parkinson’s disease. *New England Journal of Medicine*.

[62] Govindarajan M., Rajeswary M. (2015). Ovicidal and adulticidal potential of leaf and seed extract of *Albizia lebbeck* (L.) Benth. (Family: fabaceae) against *Culex quinquefasciatus, Aedes aegypti*, and *Anopheles stephensi* (Diptera: Culicidae). *Parasitology Research*.

[63] Besra S. E., Gomes A., Chaudhury L., Vedasiromoni J. R., Ganguly D. K. (2002). Antidiarrhoeal activity of seed extract of *Albizzia lebbeck* Benth. *Phytotherapy Research*.

